# Cashew apple juice supplementation enhances leukocyte count by reducing oxidative stress after high-intensity exercise in trained and untrained men

**DOI:** 10.1186/s12970-019-0299-2

**Published:** 2019-07-31

**Authors:** Piyapong Prasertsri, Thapanee Roengrit, Yupaporn Kanpetta, Terdthai Tong-un, Supaporn Muchimapura, Jintanaporn Wattanathorn, Naruemon Leelayuwat

**Affiliations:** 1 Faculty of Allied Health Sciences, Burapha University, Chonburi, 20131 Thailand; 2 Department of Basic Medical Science, Faculty of Medicine Vajira Hospital, Navamindradhiraj University, Bangkok, 10300 Thailand; 3 Faculty of Science, Buriram Rajabhat University, Buriram, 31000 Thailand; 4 Department of Physiology, Faculty of Medicine, Khon Kaen University, Khon Kaen, 40002 Thailand; 5 Exercise and Nutrition Sciences and Innovation Research Unit, Burapha University, Chonburi, 20131 Thailand; 6 Exercise and Sport Sciences Development and Research Group, Khon Kaen University, Khon Kaen, 40002 Thailand

**Keywords:** Cashew, Leukocyte, Oxidative stress, Vitamin C, Exercise, Training

## Abstract

**Background:**

Cashew apple juice (CAJ) was shown to improve immunological mechanisms by regulating a balance between reactive oxygen species and antioxidant concentrations. However, no study exploring the effects of the CAJ and training status on the immune system and oxidative stress induced by exercise. Therefore, we investigated the effects of CAJ supplementation primarily on leukocyte counts and secondary on oxidative stress and cortisol changes after high-intensity exercise in trained and untrained men.

**Methods:**

Ten moderately (endurance) trained (Age = 21.5 ± 0.97 yr., VO2max = 45.6 ± 4.12 mL/kgBM/min) and ten sedentary men (Age = 20.4 ± 2.72 yr., VO2peak = 32.2 ± 7.26 mL/kgBM/min) were randomized to ingest either daily CAJ or a placebo at 3.5 mL/kgBM/day for 4 weeks, with a four-week washout period. Before and after each period, they performed 20-min, high-intensity cycling (85% VO2max), with blood samples collected immediately preceding and the following exercise. Samples were analyzed to determine leukocyte counts, malondialdehyde, 8-isoprostane, and cortisol concentrations. A repeated measures analysis of variance was used to examine the effects of supplement and training status over time with an alpha level of 0.05.

**Results:**

There was no interaction between supplement and training status on those variables before and after exercise. However, CAJ raised resting neutrophil counts and exercise-induced leukocyte counts in the trained group (all *p* < 0.05). Besides, CAJ significantly reduced plasma malondialdehyde concentrations at rest and after exercise and reduced the post-exercise plasma 8-isoprostane concentration in both groups of subjects (*p* < 0.05). Moreover, CAJ reduced plasma cortisol after exercise in the untrained subjects.

**Conclusions:**

We suggest that 4-week CAJ supplementation can enhance exercise-induced leukocyte and resting neutrophil counts in trained men. The possible mechanism is a reduction in oxidative stress. However, the supplementation did not change the immune responses of untrained men, but it did reduce stress hormone concentrations.

**Trial registration number:**

TCTR20181127002 Registered 26 November 2018 “retrospectively registered”.

## Introduction

High-intensity aerobic training has been shown to suppress leukocyte counts in moderately fit athletes [1]. There are two possible mechanisms explaining the exercise-induced attenuation of leukocyte counts, including increased oxidative stress and stress hormone concentrations (e.g., cortisol and catecholamines [2–4]). Appropriate nutritional supplementation might prevent, or help to offset, those immunosuppressive effects during high-load endurance training, especially when exposed to more extreme environments. Besides, such supplementation could be beneficial to sedentary individuals who perform less-frequent, high-intensity exercise. However, there is currently insufficient research regarding the efficacy of nutritional supplements.

Cashew apple juice (CAJ), a product of cashew manufacturing, has been reported to improve immunological mechanisms by regulating balance between reactive oxygen species and antioxidant concentrations in mice [5]. Previous studies confirmed that four- and 12-week-consumption of CAJ elicited antioxidant effects in both untrained and trained subjects [6, 7]. That effect may have been due to its essential nutritional components, including vitamin C and anacardic acids, which have established antioxidant activity [8, 9]. Moreover, the link between antioxidant supplementation and reductions in plasma cortisol concentration following strenuous exercise have now been established [10, 11]. Indeed, it is possible that CAJ acts to attenuate the immunosuppressive effect following both overload training and a single bout of high-intensity exercise in sedentary individuals. Besides, CAJ supplementation was shown by our previous study to increase endurance and strength performance [6]. The improved performances may be due to the improved immune system, oxidative stress and stress hormone. However, the chronic effect of CAJ ingestion on the exercise-induced leukocyte counts, oxidative stress and circulating cortisol in trained and untrained populations remain unknown.

Therefore, we investigated the chronic effects of CAJ supplementation on primarily on leukocyte counts and secondary on oxidative stress and cortisol changes in both endurance-trained and sedentary subjects before, and after, a bout of high-intensity, endurance exercise. Our aim was to explore possible advantageous effects of CAJ supplementation, and, if present, to identify the possible mechanisms underlying those effects. It was hypothesized that CAJ supplementation would enhance leukocyte counts, and that such a change would be associated with an attenuation of oxidative stress and circulating cortisol concentrations. Finally, it was postulated that those effects would be positively influenced by endurance training status.

## Methods

### Study design

A randomized, double-blind, crossover design was used for this experiment, with two treatment arms; CAJ supplementation and placebo (PLA). Ten moderately (endurance) trained and untrained men were randomized to ingest either daily CAJ or a placebo at 3.5 mL/kgBM/day for four weeks, with a four-week wash out period. Before and after each period, they performed 20-min, high-intensity cycling (85% VO_2max_). All subjects were informed both verbally, and in writing, as to the possible risks of the study before signing a consent form. All experimental procedures were approved by the Human Ethical Committee of Khon Kaen University (Thailand; HE531365), and followed the ethical guidelines of the most recent Declaration of Helsinki (Edinburgh, 2000). This study was also retrospectively registered in the Thai Clinical Trials Registry (TCTR; identification number TCTR20181127002). Based on the observations of Özaslan et al. (2004) [12], in which vitamin C supplementation (4 mg/day for four weeks) increased leukocyte counts by 7.3% compared to a control group, power calculations (80% power and alpha level of 0.05) were used to determine the required sample size to observe a 5% increase in leukocyte counts. Accordingly, the sample size for this study was ten subjects for each of the two experimental groups (endurance-trained and sedentary).

### Subjects

Ten moderately trained men and ten untrained men in Khon Kaen province undertook this study (Table 1). Subjects were recruited during January 2012–December 2012. Before enrolling, all subjects were examined general health, including blood chemistry, health questionnaires, and physical examinations. This was a single-gender investigation, since gender influences on oxidative stress and the inflammatory response to exercise have been reported. Both population samples were of a similar age and of normal body mass for Thai adults since both factors affect oxidative stress biomarkers and antioxidant responses to exercise [13]. The following inclusion criteria were applied to both groups: 1) an absence of any health-related conditions 2) no use of any nutritional supplements for at least 6 months, but also during this study, c) non-smokers or drinkers. Finally, the untrained subjects had a sedentary lifestyle, and did not perform exercise of any form more than twice per week, while trained subjects performed moderate- to high-intensity endurance exercise at least six days per week for at least the previous two years.

**Table 1:** Baseline physical and physiological characteristics of subjects

	Untrained group (*n* = 10)	Trained group (*n* = 10)	*P* value
Age (yr)	20.4 ± 2.72	21.5 ± 0.97	0.24
Body mass (kg)	62.7 ± 8.30	67.2 ± 10.18	0.29
Height (m)	1.69 ± 0.06	1.70 ± 0.07	0.36
BMI (kg/m^2^)	21.9 ± 1.73	22.7 ± 2.35	0.38
Waist circumference (cm)	73.6 ± 6.87	75.6 ± 4.27	0.43
Hip circumference (cm)	92.2 ± 5.05	92.3 ± 4.96	0.97
W/H ratio	0.80 ± 0.04	0.80 ± 0.03	0.19
Body fat (%)	21.9 ± 8.09	16.1 ± 6.58	0.10
Fat mass (kg)	14.3 ± 5.61	13.8 ± 8.28	0.88
Fat free mass (kg)	48.9 ± 5.68	50.8 ± 4.95	0.43
V̇O_2,peak_ (ml/kgBM/min) V̇O_2,max_ (ml/kgBM/min)	32.2 ± 7.26	45.6 ± 4.12	0.00
V̇O_2,peak_ (ml/kgFFM/min) V̇O_2,max_ (ml/kgFFM/min)	42.2 ± 7.69	58.5 ± 4.86	0.00
Work rate_max_ (watts)	136 ± 14.30	177 ± 13.37	0.00

Values are mean ± SD, *n* = 10 in each group. *BM* body mass, *BMI* body mass index, *W/H* waist to hip circumference, *V̇O*_*2,peak*_ peak oxygen consumption, *V̇O*_*2,max*_ maximal oxygen consumption, *FFM* fat free mass, *Work rate*_*max*_ maximal work rate

*P* value < 0.05, significantly different from the untrained group

### Baseline measurements

Baseline data were collected prior to commencing supplementation (Table 1), including routine medical examinations and measurements of body height, mass, body mass index (BMI), and body composition. Fat distribution was estimated from the ratio of waist and hip circumferences. Body composition (excluding cranium) was assessed using dual emission X-ray absorptiometry (DXA; Lunar Prodigy whole-body scanner, GE Medical System, USA).

### Supplementation protocol

Two treatments were used within each of the population samples: an experimental CAJ supplementation and a control PLA supplementation. The CAJ supplement (Srisupphaluck Orchid Co., Ltd., Phuket, Thailand) was composed of vitamin C (3.36 mg/100 g), branched-chain amino acids of leucine (1.64 mg/100 g), isoleucine (3.04 mg/100 g), and valine (0.19 mg/100 g), and contained a total sugar content of 69.8 g/100 mL) [6]. The PLA was prepared with a total sugar equal to that of the CAJ. Both supplements were provided at a body-mass dependent concentration of 3.5 mL/kg/day.

### Randomization

**Fig. 1:**
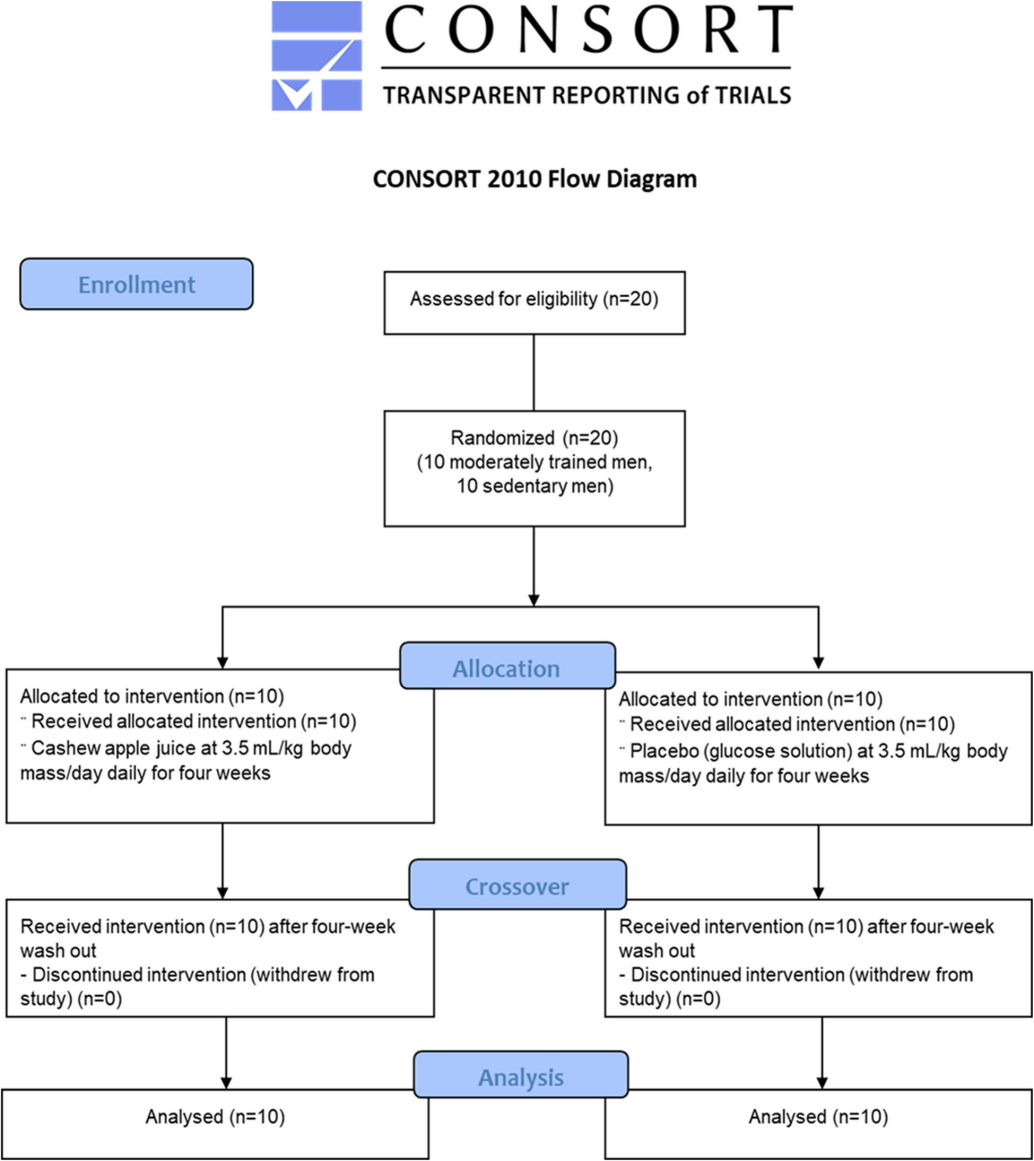
Flow chart of the trial protocol

Within each of the subject groups (1:1), an electronic randomization procedure was used to assign an equal number of participants into either the experimental or control supplementation groups (Fig. 1). Each number was kept in a sealed envelope before the allocation. A researcher who generated the random allocation sequence was different from the one who enrolled participants, and assigned participants to interventions**.**

### Study protocol

Prior to commencing supplementation, all subjects performed an incremental exercise test [6] to volitional exhaustion, thereby permitting the determination of peak oxygen consumption (V̇O_2,peak_) and maximal work rate for every participant (Table 1). One week later, they commenced the 12-week nutritional supplementation phase, commencing with four weeks of supplement administration, followed by a washout period (four weeks) before entering the second arm of supplementation. Immediately before and after supplementation, each participant performed a 20-min bout of high-intensity cycling (Corival, Lode B.V., The Netherlands), the intensity of which was set at 85% of each person’s maximal work rate. Expired-gas samples were collected throughout (PowerLab 8/30 AD Instruments, Australia), as were oxygen saturation, heart rate, and electrocardiographic data (Nihon Kohden Monitoring System Network, Japan). Venous blood samples (12 mL) were taken from the antecubital vein and collected into EDTA-treated and lithium heparin-treated tubes. Those samples were collected before and after this exercise bout, and used to analyze leukocyte counts and the concentrations of plasma 8-isoprostane, malondialdehyde, and serum cortisol. Whole blood for leukocyte counts remained capped at an ambient temperature was analysed within four hours. Room temperature was 24 ± 2 °C and humidity during testing was 59 ± 8%. All tests were performed at the same time of the day.

Subjects were instructed not to alter their diets or physical activities all over the course of the entire 12 weeks of this experiment. One week before beginning and finishing the experiment, subjects were asked to record their daily diet and physical activity patterns for three days of that week; two days from the middle of the week and one weekend day.

### Analysis of leukocyte counts and serum cortisol concentration

Whole blood sample for three mL in an EDTA-treated tube was analyzed for the counts of total leukocyte, monocyte, neutrophil, and lymphocyte using the routine hematology laboratory method (XT-2000i Automated Hematology Analyzer, Meditop Inc., Bangkok, Thailand) in Srinagarind hospital, Faculty of medicine, Khon Kaen University. The Automated Hematology Analyzer was calibrated before every measurement.

Blood sample for three mL in a lithium heparin-treated tube was analyzed for serum cortisol concentration using the radioimmunoassay technique in Srinagarind hospital, Faculty of medicine, Khon Kaen University. The assay is based on the competition between the labelled cortisol and cortisol contained in calibrators or specimens to be assayed for a fixed and limited number of antibody binding sites bound to the solid phase. After incubation, the unbound tracer is easily removed by a washing step. The amount of labelled cortisol bound to the antibody is inversely related to the amount of unlabelled cortisol initially present in the sample.

### Analysis of plasma MDA and 8-isoprostane concentrations

Blood samples (3 mL) in the EDTA-treated tubes were centrifuged at 4 °C and 2,500 *g* for 15 min to remove red blood cells and to recover plasma. The concentration of plasma MDA was analyzed indirectly from the concentration of Thiobarbituric acid reactive substances using Draper’s method [14]. The basis of the TBA methods is the reaction of MDA with TBA at low pH and high temperature to form a colored complex, the MDA-TBA complex, with an absorption maximum at 532–535 nm that can be measured by visible absorption spectrophotometry.

The concentration of plasma 8-isoprostane was analyzed using 8-isoprostane EIA kit (Cayman Chemical Company, USA). The competition between 8-isoprostane and 8-isoprostane acetylcholinesterase (AChE) conjugate for specific antiserum binding sites. The product of the enzymatic reaction has yellow colour and absorb at 412 nm. The intensity of colour measured by spectrophotometer. The amount of free 8-isoprostane show as inversely proportional with intensity.

### Statistical analyses

Data were tested for normal distribution (Shapiro-Wilks), homogeneity of variance (Levene’s test of equality of error variance), and sphericity (Mauchly’s). Data were then compared between the CAJ and the PLA supplements, and between the trained and untrained subjects using two-way analysis of variance (repeated measures). The data were initially compared prior to commencing the supplements using a one-way analysis of variance (ANOVA) with an *alpha* level of 0.05 to ensure there was no significant treatment order effect of supplement. A two-way analysis of variance (ANOVA) with an *alpha* level of 0.05 was run to evaluate the interaction effects of nutritional supplementation and the endurance training status at rest and after exercise, before and after the supplementation. When significant interactions were observed, post hoc analysis was performed using Tukey test comparison. Effect sizes were reported as partial Cohen’s d with the following effect thresholds: small (d = 0.2), medium (d = 0.6) and large (d = 1.2). All data are shown as mean with standard deviations (±). Probabilities at the 5% level were considered as statistically significant. All statistics were evaluated using SPSS Statistics for Windows (IBM Inc., Armonk, NY, USA).

## Results

### Pre-experimental status

Prior to commencing this experiment, the aerobic power of the two population samples differed significantly, regardless of fat-free mass or total-body mass (*p* < 0.05; Table 1). There were no significant differences in baseline anthropometrics, body composition, or physiological characteristics between the subject groups (*p* > 0.05; Table 1). Similarly, the baseline immunological variables and cell counts did not differ, and were within the reference intervals for people of normal health. Furthermore, we did not find any treatment order effect on all variables in both untrained and trained subjects (*p* > 0.05). Furthermore, no harmful effect from the experiment to all subjects.

### Leukocyte counts at rest and immediately after exercise

**Fig. 2:**
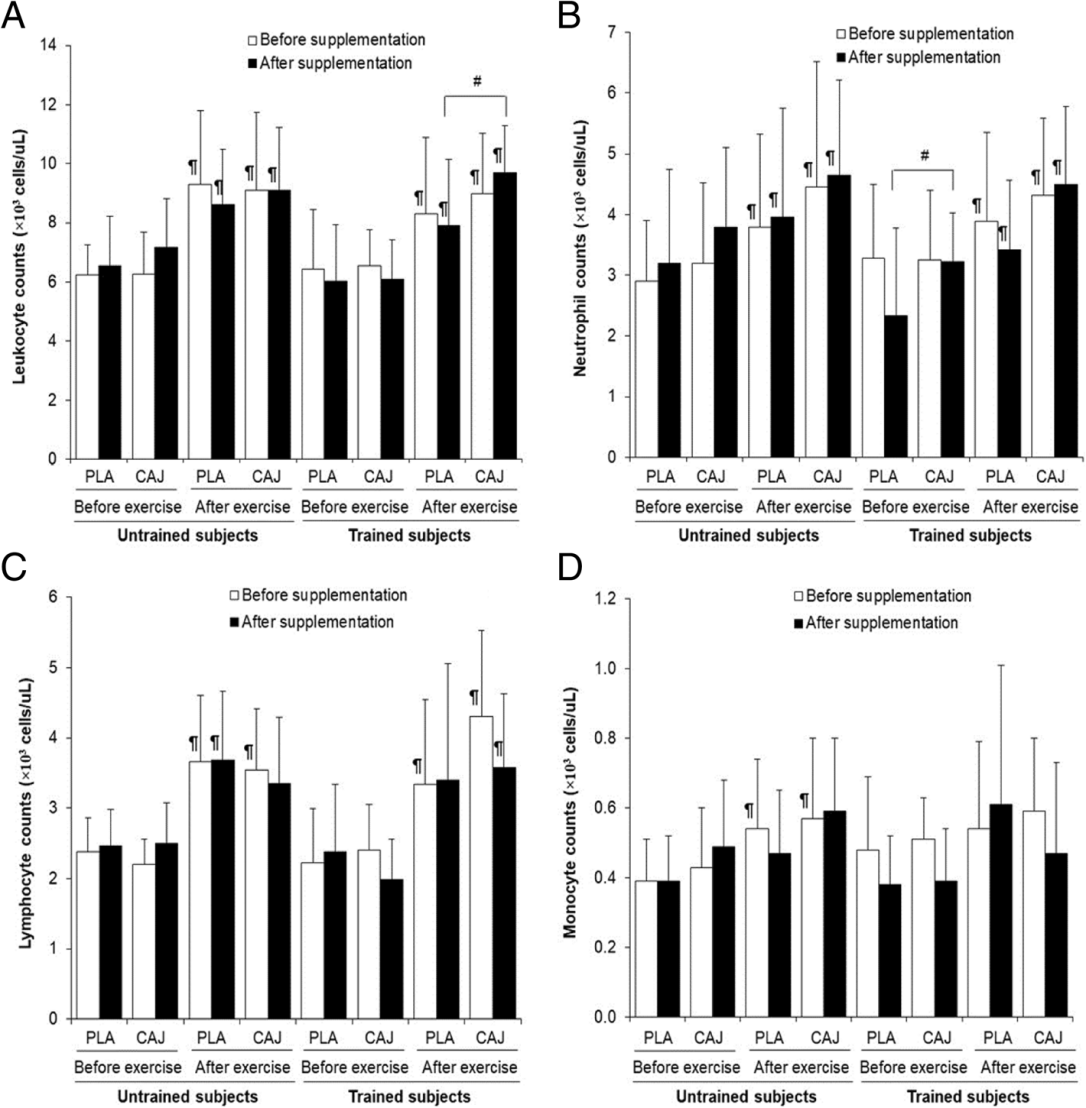
Total leukocyte 1(**a**), neutrophil 1(**b**), lymphocyte 1(**c**), and monocyte 1(**d**) counts before and immediately after exercise at 85% V̇O_2,peak_ or 85% V̇O_2,max_ after four-week placebo (PLA) and cashew apple juice (CAJ) supplementation. Values are mean ± SE, n = 10 in each group. ^#^ significantly different from PLA supplementation at the same condition, *p* < 0.05, ¶ significantly different from before exercise at the same condition, *p* < 0.05

There was a significant main effect of supplementation (F = 6.77, *p* = 0.02, effect size = 0.29), with the CAJ supplement resulting in a greater total leukocyte counts immediately after exercise (Fig. 2a; CAJ vs PLA: 9.44 ± 1.41 vs 7.91 ± 2.23 × 10^3^ cells/μL; mean difference = 1.53, 95% CI = − 2.83, − 0.24, *p* = 0.03). Furthermore, there was a significant supplement effect on the resting neutrophil counts (F = 11.25, *p* = 0.00, effect size = 0.40). In this case, CAJ significantly produced increased resting neutrophil counts (Fig. 2b; CAJ vs PLA: 3.23 ± 0.84 vs 2.34 ± 1.43 × 10^3^ cells/μL; mean difference = 0.89, 95% CI = − 1.66, − 1.26, *p* = 0.03). However, neither CAJ nor PLA modified lymphocyte (Fig. 2c) or monocyte counts (Fig. 2d) in either subject group, either at rest or immediately after exercise. Finally, there was no interaction between supplement and training status over time for the leukocyte counts (total leukocyte: F = 0.27, *p* = 0.61; neutrophil: F = 0.59, *p* = 0.45; monocyte: F = 2.07, *p* = 0.17; lymphocyte: F = 0.00, *p* = 0.97). Both trained and untrained groups had increased leukocyte, neutrophil, and lymphocyte counts after exercise compared with before exercise in both supplement groups (*p* < 0.05). Only monocyte counts before both supplementations in untrained subjects was increased after exercise compared with before exercise (*p* < 0.05).

### Oxidative stress at rest and immediately after exercise

**Fig. 3:**
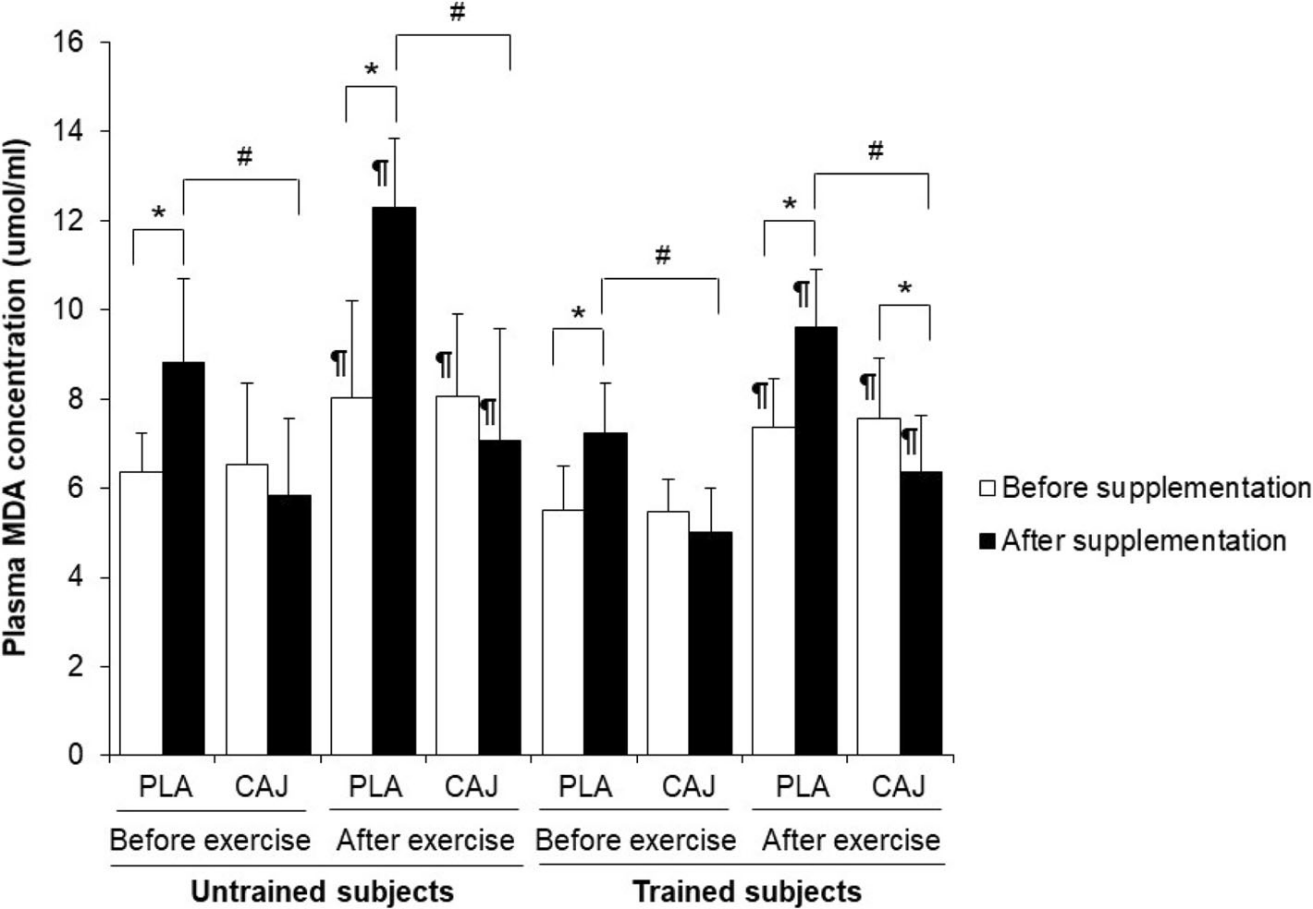
Plasma MDA concentrations before and immediately after exercise at 85% V̇O_2,peak_ or 85% V̇O_2,max_ after four-week placebo (PLA) and cashew apple juice (CAJ) supplementation. Values are mean ± SD, n = 10 in each group. MDA, malaondialdehyde. * Significantly different from before supplementation, *p* < 0.05, ^#^ significantly different from PLA supplementation, *p* < 0.05, ^@^ significantly different from untrained group, *p* < 0.05. ¶ significantly different from before exercise at the same condition, *p* < 0.05

**Fig. 4:**
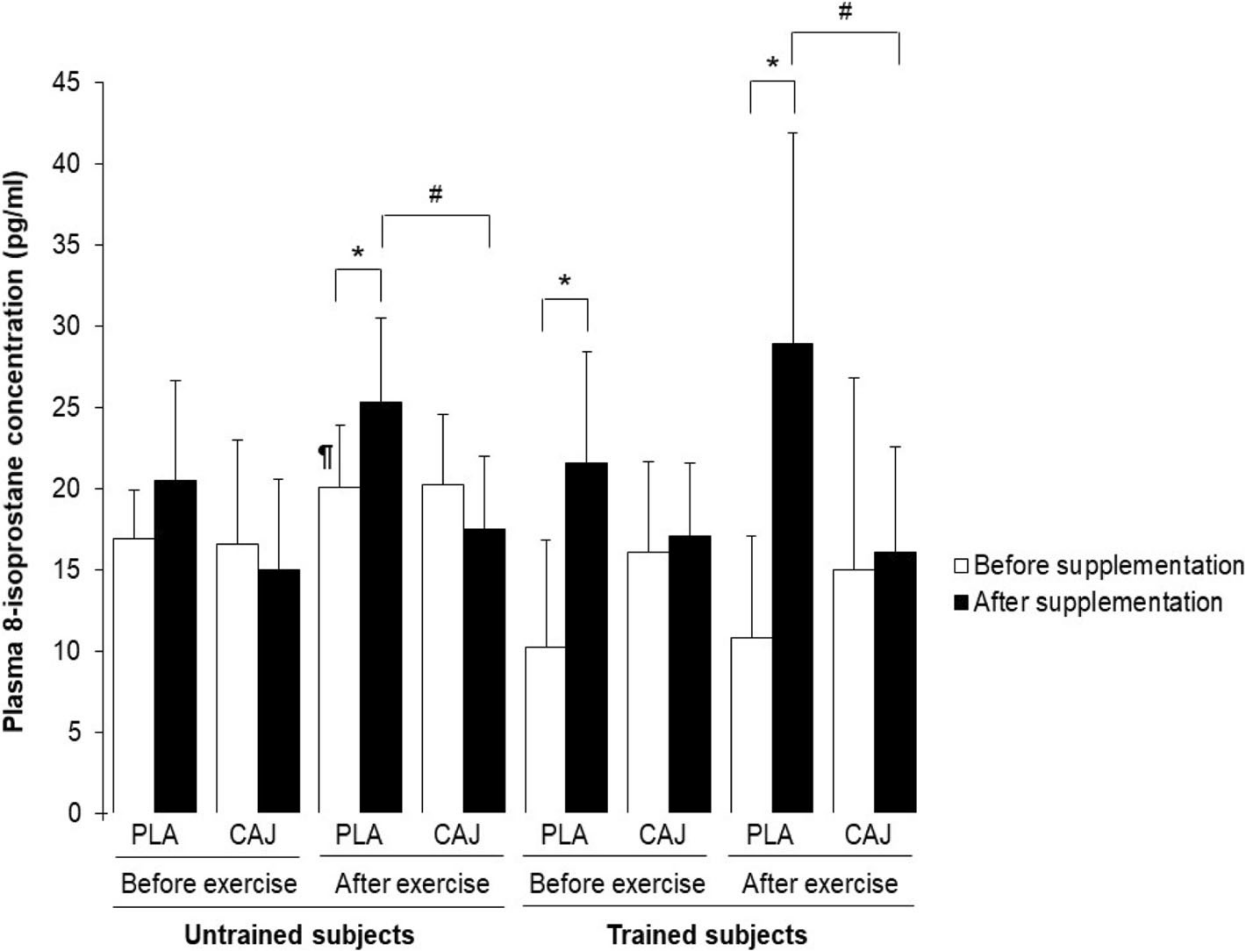
Plasma 8-isoprostane concentrations before and immediately after exercise at 85% V̇O_2,peak_ or 85% V̇O_2,max_ after 4-week placebo (PLA) and cashew apple juice (CAJ) supplementation. Values are mean ± SD, *n* = 10 in each group. * Significantly different from before supplementation, *p* < 0.05, ^#^ significantly different from PLA supplementation, *p* < 0.05. ¶ significantly different from before exercise at the same condition, *p* < 0.05

There was no effect of training status on any of the oxidative stress markers, either before or after supplementation (all *p* > 0.05). Similarly, there was no interaction between the supplementation and training status of the two groups in oxidative stress markers (Figs. 3 and 4; malondialdehyde and 8-isoprostane concentrations; F = 0.61, *p* = 0.45), either before or following supplementation.

A significant main (supplement) effect was observed on resting MDA concentration (Fig. 3; F = 117.12, *p* = 0.00, effect size = 0.87). For the resting (pre-exercise) MDA concentrations, there were significantly lower plasma MDA concentrations in both untrained and trained groups following CAJ supplementation, relative to PLA supplementation at the same point of the experiment (Fig. 3; CAJ vs PLA: 5.85 ± 1.71 vs 8.83 ± 1.88 μmol/mL in untrained group; mean difference = 2.99, 95% CI = 1.42–4.56, *p* = 0.00; and 5.00 ± 1.02 vs 7.22 ± 1.14 μmol/mL in trained group; mean difference = 2.23, 95% CI = 1.17–3.28, *p* = 0.00). After PLA supplementation, and when compared with the pre- supplementation status, resting MDA concentrations were increased in both trained and untrained groups (*p* < 0.05).

Following an acute exercise bout, and after the four-week supplementation period, a significant supplement effect on plasma MDA concentrations was observed (F = 117.12, *p* = 0.00, effect size = 0.87). Immediately after exercise, plasma MDA concentrations in both untrained and trained groups were significantly increased but lower when those subjects had been taking the CAJ supplementation, relative to values observed following the PLA (Fig. 3; CAJ vs PLA: 7.07 ± 2.50 vs 12.28 ± 1.57 μmol/mL in untrained group; mean difference = 5.21, 95% CI = 3.50–6.91, *p* = 0.00; and 6.37 ± 1.25 vs 9.60 ± 1.29 μmol/mL in trained group; mean difference = 3.23, 95% CI = 1.95–4.51, *p* = 0.00).

A significant supplementation effect was observed on plasma 8-isoprostane concentration immediately after acute exercise (F = 18.18, *p* = 0.00, effect size = 0.50). Following that exercise, there were significantly lower plasma 8-isoprostane concentrations in both untrained and trained groups, when those subjects had been taking the CAJ supplementation, relative to the PLA observations from the same time point (Fig. 4; CAJ vs PLA: 17.51 ± 4.50 vs 25.37 ± 5.17 ρg/mL in untrained group; mean difference = 7.86, 95% CI = 3.62–12.10, *p* = 0.00; and 16.09 ± 6.53 vs 28.92 ± 12.98 ρg/mL in trained group; mean difference = 12.83, 95% CI = 3.62–22.04, *p* = 0.01). However, immediately after the acute exercise bout, plasma 8-isoprostane concentrations were increased during PLA supplementation in untrained group (*p* < 0.05).

### Cortisol concentrations at rest and immediately after exercise

**Fig. 5:**
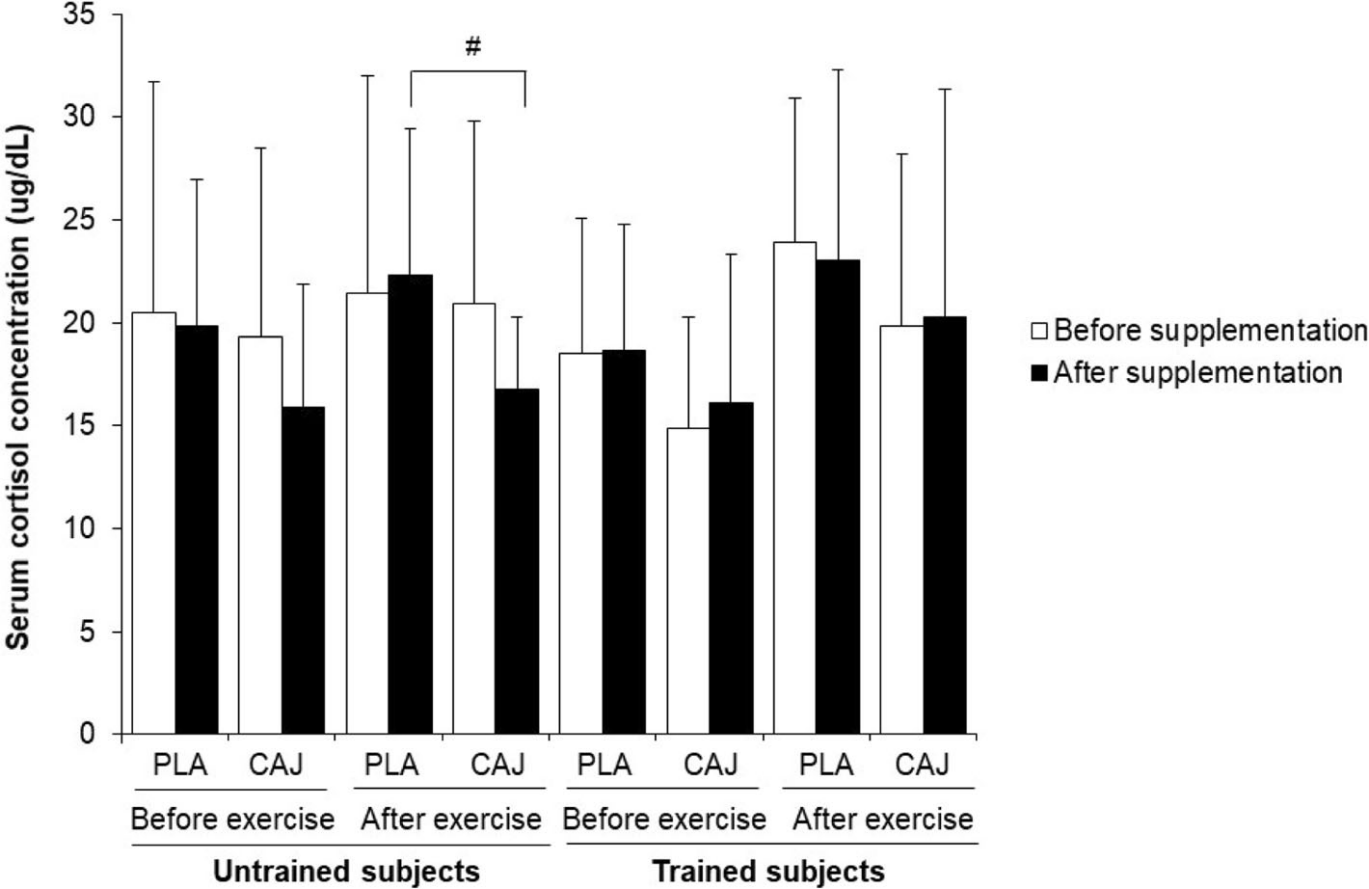
Plasma cortisol concentrations before and immediately after exercise at 85% V̇O_2,peak_ or 85% V̇O_2,max_ after four-week placebo (PLA) and cashew apple juice (CAJ) supplementation. Values are mean ± SD, n = 10 in each group. ^#^ significantly different from PLA supplementation, *p* < 0.05

The concentrations of resting cortisol before exercise were not different between the CAJ and PLA supplementations in untrained and trained groups (Fig. 5; *p* > 0.05). There was also no interaction effect between either of these supplements and training status of the two population samples on cortisol concentration (F = 0.13, *p* = 0.73), either before and after supplementation. There was, however, a significant supplementation effect on serum cortisol concentrations immediately after acute exercise (F = 10.37, *p* = 0.01, effect size = 0.39). Immediately after exercise, plasma cortisol concentrations in both untrained and trained group were not significantly changed from before exercise (*p* < 0.05). However, in this condition cortisol concentrations in untrained group were significantly lower when they had been taking the CAJ supplement, relative to PLA supplement (Fig. 5; CAJ vs PLA: 16.77 ± 3.48 vs 22.29 ± 7.16 μg/dL; mean difference = 5.52, 95% CI = 0.59–10.45, *p* = 0.03), but there was no change observed in endurance-trained group (*p* > 0.05).

## Discussion

The novel findings of the current study were that CAJ supplementation for four weeks increased leukocyte counts, while simultaneously decreasing oxidative stress, following an acute bout of high-intensity exercise in trained men. Furthermore, the CAJ supplementation increased neutrophil counts while simultaneously reducing oxidative stress and stress hormone concentrations in untrained men. These antioxidant effects following exercise were observed in both endurance-trained and untrained men. Furthermore, CAJ supplementation even decreased oxidative stress at rest. Since there was no apparent influence of endurance training status, that is, both groups of subjects revealed similar results, then it is concluded that CAJ supplementation is beneficial to men, both in resting states and in response to an acute bout of high-intensity aerobic exercise. It remains uncertain whether these effects will also occur in women.

These results partially supported our main hypotheses concerning the enhancement of leukocyte and neutrophil counts in response to high-intensity endurance exercise in trained men, although there were no changes in the other leukocyte classes, such as monocytes and lymphocytes. This seems to show that CAJ supplementation has a positive effect of immunosuppression. Our secondary hypotheses, in which mechanisms of immunosuppression were postulated, was also supported. That is, there was reduced oxidative stress, which was brought about by attenuating lipid peroxidation and stress hormone (cortisol) concentrations. The reduction in lipid peroxidation as indicated by decreasing concentrations of MDA and plasma 8-isoprostane, a high-specific biomarker.

The increased exercise-induced leukocyte counts after four-week CAJ supplementation seem to show the pro-inflammatory effect, which is essential to growing muscles strength. Exercise-induced micro-tears cause the inflammation in the muscles which must be repaired and rebuilt. Although leukocyte counts are in the normal range in the trained and untrained subjects in this study, which may be due to their moderate training, which may be not severe enough, the increased leukocyte counts after the high-intensity exercise may be applied for the highly trained individuals who suffered from the immunosuppression. We have a limitation for this point. Besides, a process of inflammation includes vasodilation induced by cytokines leading to increased blood flow and immune cell to the injured site. The vasodilation in this study is supported by the increased nitric oxide, which is vasodilator in trained subjects.

Antioxidants are essential for sustaining an efficient immune response [15–17], and vitamin C are likely to support the immune system in combatting oxidative stress [18]. CAJ has been reported to improve immunological mechanisms by regulating the balance between reactive oxygen species and antioxidant concentrations in mice [5]. Previous studies confirmed that four- and 12-week-consumption of CAJ elicited antioxidant effects in both untrained and trained subjects [6, 7]. The CAJ supplement contained a significant vitamin C content (3.36 mg/100 g), and although evidence of an efficacious influence from exogenous antioxidant vitamin C on immune function during exercise is limited, some groups have shown that vitamin C helps to strengthen and protect the immune system [17, 19].

The role of vitamin C is counteracting ROS proliferation induced by the high-intensity exercise [19], is believed to occur through helping maintain the redox integrity of the immune cells [17]. It has also been reported that vitamin C stimulates antibody and immune system cellular activity, such as that of the phagocytes and neutrophils [20]. Vitamin C supplementation seems to enhance lymphocyte proliferation, neutrophil chemotaxis, and phagocytosis of phagocytes, and thereby promote microbial killing [18]. Besides, vitamin C also acts as an essential factor for developing the maturation of T cells, which are a class of lymphocytes [21].

Therefore, on its own, vitamin C should prove beneficial. However, the CAJ also contains another antioxidant, the anacardic acids. The presence of anacardic acids in the CAJ may, in addition to the effect of vitamin C, help prevent the generation of superoxide radicals by inhibiting xanthine oxidase, and by increasing heme oxygenase-1, which is an antioxidant enzyme within the immune system [22]. Overall, the combined antioxidant content of CAJ may act synergistically to reduce oxidative stress and improve leukocyte counts, both at rest and during an acute bout of high-intensity exercise. The results of the current experiment open the possibility that supplementation with CAJ has the potential to enhance immune function, and to reduce the risk of infections and illness that are sometimes experienced following acute bouts of high-intensity endurance exercise and training [19].

The changes in the immune cell counts may be attributed to decreases in lipid peroxidation. The decrease in plasma 8-isoprostane and MDA concentrations resulting from CAJ supplementation observed in this study are in agreement with previous studies [23, 24]. Previous research from the current group demonstrated a significant increase in plasma vitamin C concentration in both trained and untrained subjects compared to a PLA supplement, following a similar four-week CAJ supplementation [6]. Those results might be explained, at least in part, by counteracting the interaction of ROS with membrane lipids, resulting in lipid peroxidation. Vitamin C can reduce the initiation of ROS so that both the initial and prolonged lipid peroxidation is reduced [25], and therefore reducing the production of 8-isoprostane and MDA. Moreover, vitamin C can react with the plasma membrane by donating electrons to the α-tocopheroxyl radical and a trans-plasma membrane oxidoreductase activity [26], leading to decreased oxidant products.

Although the current CAJ supplementation did not improve the leukocyte counts in untrained men, it did reduce the exercise-induced secretion of cortisol. It is postulated that more prolonged supplementation with CAJ may be beneficial to the immune system by counteracting stress hormone release during heavy exercise. The reduction of cortisol concentration accompanying CAJ supplementation after training in untrained in this study was consistent with previous studies that have shown that antioxidants, and in particular vitamin C, can attenuate the rise in circulating cortisol in response to exercise [10, 11]. For instance, Peters et al. [10, 11] showed a significant reduction in post-exercise plasma cortisol in participants supplemented with 1,000–1,500 mg vitamin C daily for seven days before exercise. Besides, Carrillo et al. [27] reported a decrease in post-exercise cortisol concentration in healthy individuals supplemented with 1,500 mg vitamin C for 12 days.

### Limitations

Apart from the changes in leukocyte and neutrophil count in this study, there may be other beneficial changes in immune functions as a result of the CAJ supplementation. It is our limitation did not measure immune function. Previous studies have found improved phagocytic activity and oxidative burst of polymorphonuclear cells following vitamin C treatment in patients with type 2 diabetes mellitus [18]. Therefore, further research on the effects of CAJ supplementation on the polymorphonuclear cells functions is needed. The gender difference was not currently investigated, so these observations cannot, at this time, be generalized to women. Besides, adding other antioxidants into the juice may help in revealing a more beneficial effect. It is worth to carry out such study since it may yield a useful drink for sports person or health promotion.

## Conclusion

From this experiment, it was found that a four-week supplementation of CAJ enhanced exercise-induced leukocyte and resting neutrophil counts in endurance-trained men. The possible mechanism for this effect is via a reduction in oxidative stress. While CAJ supplementation did not significantly modify leukocyte counts in the untrained individuals, it did reduce their oxidative stress and stress hormone responses. Consequently, it is concluded that CAJ supplementation is beneficial to men, both in resting states and in response to an acute bout to high-intensity aerobic exercise.

## Data Availability

Data are presented in the manuscript, further information available upon request.

## Abbreviations

BM: Body mass
BMI: Body mass index
CAJ: Cashew apple juice
MDA: Malondialdehyde
PLA: Placebo
ROS: Reactive oxygen species
SD: Standard deviation
SEM: Standard error of the mean
V̇O_2,max_: Maximal oxygen consumption
V̇O_2,peak_: Peak oxygen consumption
